# Outcomes of and factors associated with the development of bronchopulmonary dysplasia with pulmonary hypertension in very low birth weight infants: A retrospective study in a medical center

**DOI:** 10.3389/fped.2023.1055439

**Published:** 2023-03-20

**Authors:** Han-Pi Chang, Reyin Lien, Shih-Ming Chu, Jainn-Jim Lin, Ming-Chou Chiang

**Affiliations:** ^1^ Division of Neonatology, Department of Pediatrics, Chang Gung Memorial Hospital and Chang Gung University College of Medicine, Taoyuan, Taiwan; ^2^ Division of Pediatric Critical Care Medicine, Department of Pediatrics, Chang Gung Memorial Hospital and Chang Gung University College of Medicine, Taoyuan, Taiwan; ^3^ Department of Pediatrics, New Taipei Municipal TuCheng Hospital, New Taipei, Taiwan; ^4^ Division of Respiratory Therapy, Chang Gung Memorial Hospital, Taoyuan, Taiwan; ^5^ Graduate Institute of Clinical Medical Sciences, Chang Gung University College of Medicine, Taoyuan, Taiwan

**Keywords:** bronchopulmonary dysplasia, pulmonary hypertension bronchopulmonary dysplasia, positive end-expiratory pressure, preterm infants, pulmonary hypertension, very low birth weight infants, associated factor

## Abstract

**Introduction:**

Bronchopulmonary dysplasia (BPD) with pulmonary hypertension (PH) leads to increased morbidity and mortality in extremely preterm infants. Recent studies have analyzed factors associated with development of PH in BPD; however, this research remains inconclusive, and controversy exists regarding the correlation between BPD and PH. This study aimed to investigate potential associated factors, clinical characteristics, and outcomes of BPD with pulmonary hypertension in very low birth weight (VLBW) preterm infants.

**Methods:**

We conducted a retrospective study, reviewing the records of infants with gestational age (GA) <32 weeks and birth weight <1,500 g admitted to a tertiary neonatal intensive care unit between January 2020 and October 2021 who were diagnosed with moderate to severe BPD. Echocardiogram was performed at the postmenstrual age of 36 weeks or before discharge. The diagnosis of PH was based on the findings of echocardiogram. Prenatal and postnatal characteristics, demographic data, treatment details, and outcomes were collected and analyzed.

**Results:**

A total of 139 VLBW infants with BPD were enrolled and divided into a PH group (*n* = 25) and a non-PH group (*n* = 114). The mean GA was 27.3 ± 2.3 weeks and the mean birth weight of infants with BPD was 927.3 ± 293.3 g. A multivariate logistic regression model revealed that a high positive end-expiratory pressure (PEEP) setting (OR: 2.105; 95% CI: 1.472–3.011; *p *< 0.001) in established BPD and surgical closure of patent ductus arteriosus (PDA; OR: 6.273; 95% CI: 1.574–24.977; *p *= 0.009) were associated with BPD–PH. Neonates with BPD who developed pulmonary hypertension remained hospitalized for longer (*p *< 0.001), received invasive mechanical ventilation support for longer (*p* < 0.001), had a higher incidence of retinopathy of prematurity (ROP; OR: 4.201; 95% CI: 1.561–11.304; *p *= 0.003), were more likely to require oxygen support at discharge (OR: 5.600; 95% CI: 2.175–14.416; *p *< 0.001), and were more likely to undergo tracheostomy (OR: 35.368; 95% CI: 4.03–310.43; *p *< 0.001).

**Conclusion:**

PDA ligation and a higher PEEP setting were associated with BPD–PH in our cohort study. Compared with VLBW infants with BPD but without PH, infants with BPD and PH were hospitalized for longer, and also had a higher incidence of oxygen support after discharge, ROP, and tracheostomy.

## 1. Introduction

Advanced care, including prenatal steroids, gentle ventilation policies, and surfactants, has improved the survival rate of infants with very low birth weight (VLBW) (1). The incidence of bronchopulmonary dysplasia (BPD) in extremely preterm infants in Asia ranges from 25% to 56% (2). BPD with pulmonary hypertension (PH) is associated with higher morbidity and mortality in premature infants. Immature lung development and postnatal oxidative damage cause vascular remodeling and pruning of the pulmonary blood vessels, leading to increased pulmonary vascular resistance (PVR) (3). Increased PVR and pulmonary arterial pressure in extremely preterm infants results in chronic respiratory morbidities, such as right ventricular (RV) failure or cor pulmonale with RV hypertrophy. The prevalence of PH associated with BPD is estimated to range from 10% to 60% (4), and the mortality rate increases from 14% to 50% in neonates with BPD (5, 6).

Many retrospective studies have analyzed the factors influencing the occurrence of BPD with PH, such as gestational diabetes mellitus (GDM), chorioamnionitis, low gestational age (GA), low birth weight, intrauterine growth restriction, prolonged ventilation support, and surgical closure of patent ductus arteriosus (PDA) (7–11). However, research on the development of PH and PH-associated complications is limited. Therefore, we aimed to investigate the associated factors, clinical characteristics, and outcomes of BPD with PH in VLBW preterm infants. Identifying these variables could help to reduce morbidity and mortality associated with BPD.

## 2. Methods

### 2.1. Patients

This retrospective study reviewed neonates with GA <32 weeks and birth weight <1,500 g who were admitted to the Linkou Chang Gung Memorial Hospital between January 2019 and October 2021. All included neonates were diagnosed with moderate to severe BPD and were divided into a PH and a non-PH group based on echocardiography performed at a postmenstrual age of 36 weeks or before discharge. Patients with congenital anomalies, congenital heart disease, or airway anomalies were excluded.

### 2.2. Data collection

Maternal characteristics, including antenatal steroid use, delivery mode, pregnancy-induced hypertension, GDM, pre-eclampsia, chorioamnionitis, and premature rupture of membranes, were reviewed using electronic medical records. Neonatal demographic data were collected and analyzed, including clinical characteristics, radiological images, treatment provided, ventilation settings, and outcomes.

### 2.3. Clinical variables

Antenatal steroid use was defined as corticosteroid use before delivery. Early-onset sepsis (EOS) was defined as blood cultures yielding bacteria within 1 week of life. In comparison, late-onset sepsis (LOS) was defined as blood cultures yielding bacteria after 1 week of life. Respiratory distress syndrome was clinically graded on the basis of radiographic findings within 1 day after birth (12), as follows: (1) grade I comprised fine homogenous and ground-glass shadowing; (2) grade II included bilateral widespread air bronchograms; (3) grade III involved confluent alveolar shadowing, and (4) grade IV comprised alveolar shadowing obscuring the cardiac border on plain radiography.

### 2.4. Definition of bronchopulmonary dysplasia

The diagnostic criteria for BPD and criteria for classification of sub-types were based on the definitions published in the summary of a workshop of the National Institute of Child Health and Human Development (NICHD) (13). BPD was classified as mild (no oxygen supplementation), moderate (FiO_2_ < 30%), or severe (FiO_2_ ≥ 30% and/or requirement for positive pressure support). The definitions of late-evolving and established BPD were based on a study by Htun et al. (14).

### 2.5. Pulmonary hypertension and echocardiographic variables

The diagnosis of PH was established *via* echocardiography performed at least 28 days after birth (over 36 weeks corrected age or before discharge). Pediatric cardiologists screened all echocardiographic examinations. The directionality of the shunt through an atrial septal defect, patent foramen ovale, or PDA was evaluated as follows: (1) left-to-right, (2) right-to-left, or (3) bidirectional. Septal flattening, RV hypertrophy, and dilatation were recorded by pediatric cardiologists. RV systolic pressure was estimated on the basis of tricuspid regurgitant jet velocity. The criteria for PH were as follows (8): (1) RV systolic pressure >40 mmHg; (2) bidirectional or right-to-left cardiac shunt; and (3) interventricular septal flattening, right ventricular hypertrophy or dilatation present if no tricuspid regurgitation shunt was revealed on echocardiography, and absence of a residual shunt, including atrial septal defect, ventricular septal defect, or PDA.

### 2.6. Statistical analysis

The chi-squared test or Fisher's exact test was used to analyze categorical variables. Continuous variables were analyzed using the independent samples *t*-test or the Mann–Whitney *U*-test. The PH and non-PH groups were compared on maternal factors and neonatal clinical characteristics and outcomes. A multivariate logistic regression model was used to analyze certain PH-associated factors, including gender, GA, application of high-frequency oscillatory ventilation (HFOV), and inhaled nitric oxide (iNO). Other factors analyzed were positive end-expiratory pressure (PEEP) setting, mean airway pressure (MAP) setting, and duration of highest PEEP setting in late-evolving and established BPD; PDA requiring surgical closure; time of PDA ligation; and EOS or LOS. Statistical significance was defined as a *p-*value of <0.05. All statistical analyses were conducted using the IBM SPSS software package, version 24 (IBM Corp., Armonk, NY, USA).

## 3. Results

The study included 139 patients with GA < 32 weeks, birth weight <1,500 g, and a diagnosis of moderate to severe BPD. We divided the patients into a PH group (*n* = 25) and a non-PH group (*n* = 114); the prevalence of PH was 17.9% (25/139) among VLBW infants with moderate to severe BPD. There were no statistically significant differences in maternal demographic characteristics between the PH and non-PH groups (Table 1).

**Table 1:** Maternal characteristics for preterm infants with bronchopulmonary dysplasia with and without pulmonary hypertension.

	Total (*n* = 139)	BPD (*n* = 114)	BPD–PH (*n* = 25)	*p-*value	Odds ratio
Maternal characteristics
Antenatal steroid (*n*, %)	112 (80.1)	90 (78.9)	22 (88)	0.408	1.956 (0.540–7.087)
Cesarean section (*n*, %)	97 (70)	83 (72.8)	14 (56)	0.097	0.475 (0.195–1.159)
Pregnancy-induced hypertension (*n*, %)	30 (21.6)	26 (22.8)	4 (16)	0.454	0.645 (0.203–2.047)
Gestational diabetes mellitus (*n*, %)	7 (5)	6 (5.3)	1 (4)	0.794	0.750 (0.086–6.521)
Oligohydraminos (*n*, %)	3 (2.2)	3 (2.6)	0	0.412	0.816 (0.754–0.884)
Polyhydraminos (*n*, %)	6 (4.3)	5 (4.4)	1 (4)	0.931	0.908 (0.101–8.133)
Preeclampsia (*n*, %)	18 (12.9)	15 (13.2)	3 (12)	0.876	0.900 (0.240–3.379)
Chorioamnionitis (*n*, %)	32 (23)	22 (19.3)	9 (36)	0.069	2.352 (0.919–6.021)
pPROM (*n*, %)	74 (53.2)	60 (52.6)	14 (56)	0.760	1.145 (0.479–2.737)

BPD, bronchopulmonary dysplasia; PH, pulmonary hypertension; pPROM, preterm premature rupture of membranes.

The mean GA was 27.3 ± 2.3 weeks, and the mean birth weight of infants with BPD was 927.3 ± 293.3 g. The PH group had shorter GAs (25.8 ± 1.7 vs. 27.6 ± 2.3, *p *= 0.038) and contained a higher proportion of male infants (80%, 20/25 vs. 56.1%, 64/114, *p *= 0.027). The PH group had a higher incidence of hemodynamically significant PDA (hsPDA; OR: 4.910; 95% CI: 1.385–17.414; *p *= 0.008), PDA ligation (OR: 6.364; 95% CI: 2.227–18.185; *p *< 0.001), later PDA ligation (*p *< 0.001), and postnatal steroid use (OR: 5.417; 95% CI: 2.145–13.679; *p* < 0.001). They were also more likely to receive HFOV (OR: 4.444; 95% CI: 1.560–12.659; *p *= 0.004) and inhaled nitric oxide (OR: 5.417; 95% CI: 2.145–13.679; *p* < 0.001), had a higher incidence of LOS (OR: 3.550; 95% CI: 1.397–9.024; *p *= 0.006), and required higher settings for PEEP (*p *< 0.001) and MAP (*p *< 0.001) in cases of established and late-evolving BPD treated with invasive or non-invasive ventilation (Table 2).

**Table 2:** Characteristics of preterm infants with bronchopulmonary dysplasia with and without pulmonary hypertension.

	Total (*n* = 139)	BPD (*n* = 114)	BPD–PH (*n* = 25)	*p-*value	Odds ratio
Neonatal characteristics
Male (*n*, %)	84 (60.4)	64 (56.1)	20 (80)	0.027*	3.125 (1.096–8.908)
Gestational age (weeks), mean ± SD	27.3 ± 2.3	27.6 ± 2.3	25.8 ± 1.7	0.038*	NA
Birth weight (g), mean ± SD	927.3 ± 293.3	962.6 ± 297.3	766.4 ± 213.5	0.08	NA
Small for gestational age (*n*, %)	18 (12.9)	15 (13.2)	3 (12)	0.876	0.900 (0.240–3.379)
EOS (*n*, %)	13 (9.3)	11 (9.6)	2 (8)	0.798	0.814 (0.169–3.925)
LOS (*n*, %)	56 (40.3)	40 (35.1)	16 (64)	0.006*	3.550 (1.397–9.024)
Ureaplasma (*n*, %)	16 (11.5)	8 (7)	8 (32)	0.058	4.000 (0.920–17.396)
RDS grade					
RDS grade I–II (*n*, %)	79 (56.8)	64 (56.1)	15 (60)	0.724	1.172 (0.485–2.830)
RDS grade III–IV (*n*, %)	60 (43.2)	50 (43.9)	10 (40)	0.724	0.853 (0.353–2.060)
Pneumothorax (*n*, %)	10 (7.2)	8 (7)	2 (8)	0.863	1.152 (0.229–5.786)
hsPDA (*n*, %)	88 (63.3)	67 (58.8)	21 (84)	0.008*	4.910 (1.385–17.414)
PDA ligation (*n*, %)	63 (45.3)	43 (37.7)	20 (80)	<0.001*	6.605 (2.310–18.886)
Time of PDA ligation (days), mean ± SD	9 ± 14	7 ± 14	14 ± 15	<0.001*	NA
HFOV (*n*, %)	74 (53.2)	54 (47.4)	20 (80)	0.004*	4.444 (1.560–12.659)
iNO (*n*, %)	32 (16.5)	19 (16.7)	13 (52)	<0.001*	5.417 (2.145–13.679)
Postnatal steroid (*n*, %)	34 (24.5)	21 (18.4)	13 (52)	<0.001*	4.798 (1.919–11.996)
Ventilator setting					
PEEP, maximum level in evolving BPD (cm H_2_O), mean ± SD	6 ± 1	6 ± 1	7 ± 1	<0.001*	NA
PEEP, maximum level in established BPD (cm H_2_O), mean ± SD	6 ± 2	6 ± 1	9 ± 2	<0.001*	NA
MAP, maximum level in evolving BPD (cm H_2_O), mean ± SD	9.9 ± 2.8	9.2 ± 2.2	13 ± 3.5	<0.001*	NA
MAP, maximum level in established BPD (cm H_2_O), mean ± SD	9.3 ± 3.6	8.4 ± 2.4	13.5 ± 5	<0.001*	NA
Duration of maximum PEEP in established BPD (days), mean ± SD	11 ± 9	11 ± 9	12 ± 7	0.34	NA
BPD, median (IQR)
Moderate (*n*, %)	15 (10.8)	13 (11.4)	2 (8)	0.619	0.676 (0.143–3.202)
Severe (*n*, %)	124 (89.2)	101 (88.6)	23 (92)	0.619	1.480 (0.312–7.016)

RDS, respiratory distress syndrome; hsPDA, hemodynamically significant patent ductus arteriosus; PDA, patent ductus arteriosus; HFOV, high-frequency oscillatory ventilation; iNO, inhaled nitric oxide; PEEP, positive end-expiratory pressure; MAP, mean airway pressure; BPD, bronchopulmonary dysplasia; PH, pulmonary hypertension; EOD, early-onset sepsis; LOS, late-onset sepsis.

*Statistically significant at *p* < 0.05.

Neonates with PH stayed in the hospital for longer (*p *< 0.001), received invasive mechanical ventilation support for longer (*p* < 0.001), and had a higher incidence of retinopathy of prematurity (ROP; OR: 4.201; 95% CI: 1.561–11.304; *p *= 0.003), provision of oxygen support at discharge (OR: 5.600; 95% CI: 2.175–14.416; *p * < 0.001), and tracheostomy (OR: 35.368; 95% CI: 4.03–310.43; *p *< 0.001) (Table 3). We assessed the mortality rate within 1 year. No mortality occurred in the non-PH group; a preterm infant with a GA of 26 weeks died at a corrected age of 3 months as a result of severe cor pulmonale.

**Table 3:** Outcomes of preterm infants with bronchopulmonary dysplasia with and without pulmonary hypertension.

	Total (*n* = 139)	BPD (*n* = 114)	BPD–PH (*n* = 25)	*p-*value	Odds ratio
Outcome
Duration of invasive mechanical ventilation (days), mean ± SD	53.4 ± 3.8	42.3 ± 30.5	105.4 ± 70.7	<0.001*	NA
Extubation age (weeks), median (IQR)	34.4 (32.6–36.5)	34.3 (32.3–36.3)	35.1 (33.5–39.6)	0.054	NA
Duration of mechanical ventilation support (days), mean ± SD	93.2 ± 47	82.2 ± 32.3	145.3 ± 67.7	<0.001*	NA
IVH (*n*, %)	43 (30.9)	31 (27.2)	12 (48)	0.056	2.741 (1.019–5.997)
ROP (*n*, %)	68 (48.9)	49 (43)	19 (76)	0.003*	4.201 (1.561–11.304)
NEC (*n*, %)	14 (10.1)	10 (8.8)	4 (16)	0.277	1.981 (0.567–6.919)
Oxygen support at discharge (*n*, %)	46 (33.1)	30 (26.3)	16 (64)	<0.001*	5.600 (2.175–14.416)
Tracheostomy (*n*, %)	7 (5)	1 (1)	6 (24)	<0.001*	35.368 (4.03–310.43)
Length of hospital stay (days), mean ± SD	115.6 ± 46.8	93.1 ± 37.8	159.5 ± 63.5	<0.001*	NA

IVH, intraventricular hemorrhage; ROP, retinopathy of prematurity; NEC, necrotizing enterocolitis; NA, not applicable; BPD, bronchopulmonary dysplasia; PH, pulmonary hypertension.

*Statistically significant at *p* < 0.05.

A multivariate logistic regression model revealed that higher PEEP settings with invasive or non-invasive ventilators in cases of established BPD (OR: 2.105; 95% CI: 1.472–3.011; *p *< 0.001) and PDA requiring surgical closure (OR: 6.273; 95% CI: 1.574–24.997; *p *= 0.009) were significantly associated with BPD–PH (Table 4).

**Table 4:** Factors associated with BPD–PH in very low birth weight infants based on multivariate logistic analysis.

Clinical characteristics	Odds ratio	95% CI	*p*-value
Gender: male	4.267	0.966–18.850	0.056
Gestational age, weeks	0.733	0.495–1.087	0.123
Application of HFOV	0.368	0.065–2.096	0.260
Application of iNO	0.559	0.091–3.432	0.530
Elevated PEEP in evolving BPD	0.549	0.242–1.247	0.152
Elevated PEEP in established BPD	2.105	1.472–3.011	<0.001*
Elevated MAP in evolving BPD	1.256	0.938–1.681	0.126
Elevated MAP in established BPD	1.152	0.820–1.617	0.415
Surgical closure of PDA	6.273	1.574–24.997	0.009*
Time of PDA ligation	0.961	0.911–1.014	0.147
LOS	2.104	0.493–8.973	0.315

BPD, bronchopulmonary dysplasia; PH, pulmonary hypertension; HFOV, high-frequency oscillatory ventilation; iNO, inhaled nitric oxide; PEEP, positive end-expiratory pressure; PDA, patent ductus arteriosus; LOS, late-onset sepsis.

*Statistically significant at *p* < 0.05.

## 4. Discussion

This retrospective study compared patients with BPD with and without PH in terms of the factors associated with PH, clinical characteristics, and outcomes. Most of the neonates (84%; 21/25) who had BPD with PH were born before 28 weeks' GA. Similar to previous reports, we found a lower average GA (27.6 ± 2.3 vs. 25.8 ± 1.7 weeks) and birth weight (962.6 ± 297.3 vs. 766.4 ± 213.5 g) in patients with BPD and PH in our study (8, 15).

Research has revealed that the factors for PH associated with BPD are multifactorial. Prenatal factors include chorioamnionitis, GDM, pregnancy-induced hypertension, preeclampsia, and oligohydramnios; postnatal factors include hsPDA, PDA requiring surgical closure, prolonged mechanical ventilation, and LOS (7, 9, 16, 17). Compared to neonates without BPD–PH, those with BPD–PH required higher PEEP settings in our cohort study. Patients with severe BPD have a higher rate of tracheobronchomalacia (18) and thus require a higher-level PEEP setting in clinical practice (19). Gentle ventilation includes a high rate, low tidal volume, and adequate PEEP through conventional mechanical ventilation or HFOV, and this approach has been regularly applied in patients with BPD (20). However, most ventilation strategies have focused on preventing BPD, thus providing insufficient evidence on the best approach to ventilating infants with severe established BPD; moreover, these strategies may not be relevant for chronic lung pathophysiology (19). Several studies have also revealed the use of various types of mechanical ventilation support and medications for neonates with severely established BPD across multiple centers and regions (21, 22), which could lead to different outcomes. The optimal administration of PEEP in extremely premature neonates with established BPD remains uncertain because randomized controlled trials are rarely conducted in these populations. Some studies have assumed that an elevated PEEP setting may impair cardiopulmonary function. Studies in a Rhesus monkey model have shown that a higher setting PEEP (15 cm H_2_O) contributes to a beneficial decrease in left ventricular preload or PVR, but decreases the cardiac index, stroke volume, and oxygen delivery (23). Polglase et al. indicated that high levels of PEEP improve oxygenation, but may also have adverse effects, such as increasing PVR and reducing pulmonary blood flow, in very premature lambs (24). Compression of the perialveolar capillaries is thought to cause high PEEP levels, increasing PVR (25). Recent studies have shown that patients who have acute respiratory distress syndrome with high PEEP have higher PVR and decreased RV contractility on echocardiography (26). Clinicians increase PEEP to recruit unventilated lung regions to improve oxygenation. However, the mean airway pressure is not even across the entire lung, and the overexpansion of localized lesions may contribute to high resistance. Reports also indicate that overdistended lesions may result in prolonged overexpansion with higher resistance, despite the high PEEP returning to low levels (24). Another factor is that a high PEEP can lead to increased RV afterload and reduced coronary arterial blood flow caused by coronary artery compression (27). The adverse effect of coronary vascular resistance can also decrease the left ventricular output, which leads to long-term degradation of cardiopulmonary function (28). Previous studies have shown that increased PVR occurs at PEEP levels >10 cm H_2_O, but have observed no difference between PEEP of 5 cm H_2_O and 5–10 cm H_2_O (26), which is considered to be attributable to the direct impact of the compression of intra-alveolar capillaries by overexpanded alveoli (29) at PEEP levels >10 cm H_2_O. Our study showed that patients with BPD–PH had higher PEEP (mean: 9 cm ± 2 cm H_2_O) at the established BPD stage; this finding conforms with those of previous studies, which have indicated that higher PEEP might cause increased PVR (30, 31). Previous studies have attempted to explain the relationship between high PEEP and PVR; however, most have been observational studies providing insufficient evidence to identify the causal relationship between PEEP and PH. High-quality evidence on appropriate ventilator strategies and cardiopulmonary function in extremely premature neonates with established BPD is limited, and well-designed prospective studies are required (32).

PDA ligation is considered to be an independent associated factor in development of BPD, particularly in the case of prophylactic surgical closure (33). Collaco et al. identified surgical ligation as a component of a PH risk score in patients with BPD (11). Surgical closure of the ductus arteriosus seems not to have the same advantages of increasing the alveolar surface and alveolar water clearance as closure by indomethacin/ibuprofen (34). Injury due to surgical ligation contributes to an increase in expression of genes involving pulmonary inflammation and a decrease in the pulmonary epithelial sodium channel, which is helpful for alveolar water clearance (35). Previous studies have demonstrated that surgical closure results in poorer respiratory outcomes than medication- (36) or transcatheter-induced PDA closure (37) in extremely low birth weight infants. Pulmonary inflammation and immature alveolar growth caused by PDA ligation may lead to poor pulmonary vascular growth and promote the development of PH. In the current study, PDA ligation was performed between postnatal days 6 and 66, and most PDA ligations were completed within 4 weeks of life. PDA ligation performed at any time during the study period was associated with BPD–PH. Nevertheless, patients requiring surgical PDA treatment are usually more ill than those requiring medication-induced closure, and the severity of the illness may also contribute to pulmonary vascular changes.

Compared with the non-PH group, neonates with BPD and PH had poor respiratory outcomes, including prolonged invasive mechanical ventilation, longer hospital stays, supplemental oxygen support at home, tracheostomy, and ROP; this finding is similar to those of previous studies (9, 11). Poor neurological outcomes and long-term growth have been observed in extremely preterm infants with BPD and PH (38). BPD–PH in neonates causes additional medical costs and a higher burden on parents compared to BPD alone (39). Hence, long-term follow-up of cardiopulmonary function and early intervention are important to improve neurological outcomes in infants with BPD–PH.

The current study has several limitations. First, it was a retrospective, observational, and single-center study that could not provide a complete evaluation of the associated factors. Second, routine echocardiography at a postmenstrual age of 36 weeks or before discharge was not performed in all extremely preterm infants, which might have caused selection bias in our cohort. Third, echocardiography was performed by different pediatric cardiologists at our institute, which might have resulted in reader bias. Fourth, we did not obtain long-term follow-up data such as growth and neurodevelopmental outcomes.

## 5. Conclusion

Higher PEEP settings and a higher incidence of surgical PDA ligation were significantly associated with BPD–PH in VLBW infants. The application of optimal PEEP in extremely preterm infants with BPD should be guided by well-designed clinical trials in the future. We hope that the identification of appropriate ventilation strategies will facilitate further research to address the care of these high-risk patients following this discussion. Compared with VLBW infants with BPD but without PH, infants with BPD and PH were hospitalized for longer, had a higher incidence of oxygen support at discharge and a higher risk of ROP, and were more likely to undergo tracheostomy. Further investigations are required to assess the long-term outcomes of PH in extremely premature infants with BPD.

## Data Availability

The raw data supporting the conclusions of this article will be made available by the authors without undue reservation.
